# MicroRNA-21 regulates breast cancer invasion partly by targeting tissue inhibitor of metalloproteinase 3 expression

**DOI:** 10.1186/1756-9966-29-29

**Published:** 2010-03-27

**Authors:** Bao Song, Chuanxi Wang, Jie Liu, Xingwu Wang, Liyan Lv, Ling Wei, Li Xie, Yan Zheng, Xianrang Song

**Affiliations:** 1 Provincial Key Laboratory of radiation oncology, Shandong Cancer Hospital & Institute, Jinan, Shandong, China; 2 Department of radiation oncology, Nanfang Hospital, The Southern Medical University, China; 3 Department of oncology, Shandong Cancer Hospital & Institute, Jinan, Shandong, China

## Abstract

**Background:**

MicroRNAs are non-coding RNA molecules that posttranscriptionally regulate expression of target genes and have been implicated in the progress of cancer proliferation, differentiation and apoptosis. The aim of this study was to determine whether microRNA-21 (miR-21), a specific microRNA implicated in multiple aspects of carcinogenesis, impacts breast cancer invasion by regulating the tissue inhibitor of metalloproteinase 3 (TIMP3) gene.

**Methods:**

miR-21 expression was investigated in 32 matched breast cancer and normal breast tissues, and in four human breast cancer cell lines, by Taqman quantitative real-time PCR. Cell invasive ability was determined by matrigel invasion assay in vitro, in cells transfected with miR-21 or anti-miR-21 oligonucleotides. In addition, the regulation of tissue inhibitor of metalloproteinase 3 (TIMP3) by miR-21 was evaluated by western blotting and luciferase assays.

**Results:**

Of the 32 paired samples analyzed, 25 breast cancer tissues displayed overexpression of miR-21 in comparison with matched normal breast epithelium. Additionally, incidence of lymph node metastasis closely correlated with miR-21 expression, suggesting a role for miR-21 in metastasis. Similarly, each of the four breast cancer cell lines analyzed overexpressed miR-21, to varied levels. Further, cells transfected with miR-21 showed significantly increased matrigel invasion compared with control cells, whereas transfection with anti-miR-21 significantly decreased cell invasion. Evaluation of TIMP3 protein levels, a peptidase involved in extarcellular matrix degredation, inversely correlated with miR-21 expression.

**Conclusion:**

As knockdown of miR-21 increased TIMP3 protein expression and luciferase reporter activity, our data suggests that miR-21 could promote invasion in breast cancer cells via its regulation of TIMP3.

## Background

MicroRNAs (miRNAs) are a class of small, noncoding RNA molecules of about 22 nucleotides in length that function as posttranscriptional gene regulators [1-3]. MiRNAs encoded in the genome are transcribed by RNA polymerase II in the nucleus, where they become cleaved by Drosha and processed by Dicer[4]. Mature miRNAs repress protein expression by imperfect base pairing with the 3'untranslated region (UTR) of target mRNA, leading to reduced translation and/or degradation of that mRNA molecule [1-3].

miRNAs regulate various biological processes, including development, differentiation, cell proliferation and apoptosis. Accumulating evidence suggests that alterations of some miRNAs expression may play a role in the development of human cancers [5-7]. While many miRNA, including let-7, miR-15 and miR-16 are down-regulated or deleted in cancers [8-10], oncogenic miRNAs are frequently overexpressed in tumors. Specifically, miR-21 is overexpressed in very diverse types of malignancy. miR-21 has been proposed to impact cancer progression by regulating the tumor suppressor gene Tropomyosin 1 (TM1) [11]. Further, the anti-proliferative effect of miR-21 inhibition [12] was inhibited by inactivation of programmed cell death 4 (PDCD4), suggesting that overexpression of miR-21 represses normal apoptotic signaling.

Endogenous inhibitors of matrix metalloproteinases (MMPs) play a critical role in extracellular matrix (ECM) homeostasis[13], and deregulated ECM remodeling contributes to cancer metastasis [14]. Recent evidence suggests that miR-21 promotes glioma [15] and cholangiocarcinoma [16] invasion by targeting MMP regulators. As tissue inhibitors of metalloproteinases (TIMPs) contain a consensus miR-21 binding site (http://targetscan.org/; http://pictar.mdc-berlin.de/; http://microRNA.org), and reduced expression of *TIMP3 *in breast cancer tissue has been associated with poor disease-free survival[17], we sought to determine the role of miR-21 in breast cancer invasion, and to identify whether miR-21-mediated invasion might be regulated via TIMP3.

## Methods

### Human tissue samples and cell lines

Human tissue samples were obtained by surgical resection from 32 patients with breast cancer, at Shandong Cancer Hospital and Institute from 2005 to 2006. All samples, including breast cancer and corresponding adjacent normal tissues, were preserved in liquid nitrogen for 30 min following resection. Informed consents were obtained from all subjects. Human breast cancer cell lines BCAP-37 (Chinese Type Culture Collection, Beijing, China), MCF-7 (American Type Culture Collection (ATCC) number: HTB-22), MDA-MB-231 (ATCC number HTB-26), and MDA-MB-435 (ATCC number HTB-129) were cultured in Dulbecco's modified Eagle's medium (DMEM, GIBCO, USA) containing 10% heat-inactivated fetal bovine serum (FBS, GIBCO), and maintained at 37°C in a humidified atmosphere of 5% CO_2_. Cells were passaged every 2-3 days to maintain exponential growth.

### qRT-PCR analysis of miRNA-21 and TIMP3 mRNA expression

Total RNA from tissue and cells were isolated using TRIzol reagent (Invitrogen) to obtain both miRNA and mRNA. For analysis of miR-21 expression, the stem-loop RT primer, real-time PCR primes and TaqMan MGB probe were designed as previously described [18]. Briefly, miRNAs were reverse transcribed into cDNAs by SuperScript II reverse transcriptase. Real-time PCR was performed using a standard TaqMan PCR protocol according to manufacturer's protocols (Applied Biosystems), and relative expression was calculated using the Δ*CT *method and normalized to the expression of *U6 *RNA. Relative levels of TIMP3 mRNA were examined by SYBR green real-time quantitative reverse transcription-PCR (qRT-PCR) (Applied Biosystems) and normalized to β-actin mRNA. The primers for TIMP3 were: forward primer 5'-AGTTACCCAGCCCTATGA-3', reverse primer 5'-GCAAAGGCTTAAACATCT-3'. All qRT-PCRs were performed in duplicate, and the data are presented as mean ± standard error of the mean (SEM).

### Oligonucleotide transfections

For miR-21 knockdown, cells were transfected with 50 nM of oligonucleotide with Lipofectamine 2000 (Invitrogen), according to the manufacturer's protocol. The sequences used were: 5'-UCAACAUCAGUCUGAUAAGCUA-3' (anti-miR-21 oligonucleotide); and 5'-CAGUACUUUUGUAGUACAA-3' (control oligonucleotide). For miR-21 overexpression, cells were transfected with a synthetic RNA duplex sequence corresponding to mature miR-21. The sequences were: 5'-UAGCUUAUCAGACUGAUGUUGA-3' (miR-21 oligonucleotide); and 5'-UUCUCCGAACGUGUCACGUTT-3' (control oligonucleotide). All oligonucleotides were synthesized by Genepharma Co. Ltd. The sequences of the control oligonucleotides were analyzed by BLAST search to exclude potential hits in the human transcriptome.

### Migration assay

BCAP-37, MCF-7, MDA-MB-231, and MDA-MB-435 cells were transfected with anti-miR-21, miR-21, or control oligonucleotide, cultured for 48 h, and transferred onto the top of matrigel-coated invasion chambers (24-well insert, 8 μm pore size; BD Biosciences) in a serum-free DMEM. DMEM containing 10% fetal calf serum was added to the lower chamber as a chemoattractant. After 20 h incubation, non-migrated cells were removed from the inner part of the insert with a cotton swab. Fixation and staining of migrated cells were performed using 0.1% crystal violet. Cells were quantified by fluorescence microscopy (100×).

### Western blot analysis

Cell lysates were prepared in lysis buffer (0.15 M NaCl,50 mM Tris-Cl(pH7.5), 2 mM EDTA, 0.5%Triton-100, 5 mM DTT, 0.2 mM PMSF, 2 μg/ml apoptinin) following 72 h transfection with either anti-miR-21 or control oligonucleotide. Protein concentrations of total cell lysates were measured by Bio-Rad Protein Assay, and 50 ug of total cell lysates per lane was separated by 10% SDS-PAGE. Immunoblotting was performed with rabbit anti-TIMP3 (1:500; Chemicon), and rabbit anti β-actin (1:500; Abcam) primary antibodies. Membranes were subsequently probed with horseradish peroxidase-conjugated secondary antibody (1:5000; Zhongshan Biotech, China), developed by chemiluminescence and exposed to X-ray film. Densitometry was performed with gel imaging system (Alphaimager 2200, Pharmacia Biotech Co. USA).

### Luciferase reporter assay

The human TIMP3 3'UTR target site was amplified by PCR using the primers 5'-TCTAGACAAGGAGGAACTTGGGTGA-3' (forward) and 5'-TCTAGAAATACAGAAGTGTCTCAGC-3' (reverse). The TIMP3 3'UTR was digested by *Xba *I, and cloned into the pGL3 luciferase vector (Promega, Madison, Wisconsin, USA) digested with the same restriction enzyme. This construct, named pGL3-TIMP3, transfected into MDA-MB-231 and MDA-MB-435 cell lines. At 5 h after transfection, cells were transfected again with 50 nM of anti-miR-21 or control oligonucleotide. Cells were lysed for luciferase activity was measured 24 h thereafter. pGL3 was cotransfected and used for normalization. Each transfection was repeated twice in triplicate.

### Statistical analysis

Statistical analysis was performed using the SPSS13.0 software. Values are expressed as mean ± SEM. Differences/correlations between groups were calculated with Student's t test, and Pearson's correlation test. P < 0.05 was defined as being significant.

## Results

### MiR-21 is overexpressed in breast cancer tissue

**Figure 1:**
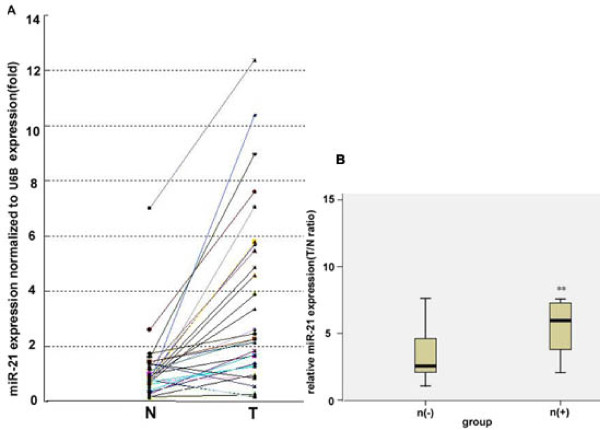
**Overexpression of miR-21 in breast cancer tissue specimens**. Total RNA was isolated from matched normal breast epithelium and breast cancer tissue using Trizol. MiR-21expression was analyzed by TaqMan quantitative real-time PCR and normalized to β-actin expression. A, Quantification of miR-21 expression in matched normal breast epithelium and breast cancer tissue surgically resected from 32 patients. N, normal tissue; T, tumor tissue. B, The ratio of miR-21expression, presented as relative T/N ratio of. The T/N ratios were analyzed statistically in patients with lymph node metastasis or without.*, P < 0.05. n, lymph node metastasis.

Matched normal breast epithelium and breast cancer tissue were obtained from 32 patients treated at Shandong Cancer Hospital and Institute from 2005 to 2006. The clinicopathologic findings of each patient are shown in Table 1. Total RNA was isolated from each sample, and miR-21 content was determined by TaqMan real-time PCR. Overexpression of miR-21 were observed in 25 of 32 cancer tissues in comparison with the matched normal tissues (Fig. 1A; *P *< 0.05), and miR-21 expression was significantly higher in patients with lymph node metastasis (Fig. 1B; *P *< 0.05).

**Table 1:** Clinicopathologic characteristics of surgically resected breast cancer speimen.

Factors	cases
Tumor size (≥ 2/<2 cm)	24/8
Histological grade (I/II~III)	7/25
Lymph node metastasis (negative/positive)	21/11
Clinical stage (I/II/III~IV)	8/17/7
ER/PR (positive/negative)	21/11
Menopausal status (yes/no)	12/20

### MiR-21 influences cell invasion of breast cancer lines

**Figure 2:**
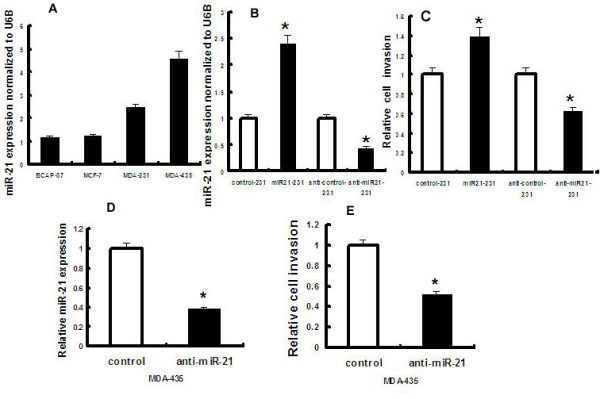
**miR-21 impacts breast cancer cell invasion in vitro**. A, Relative miR-21expression was analyzed by Taqman PCR in four breast cancer cells. B, MDA-231 cells were transfected with miR21, anti-miR-21 or appropriate control oligonucleotides. Total RNA was isolated and analysed for miR-21 expression as in A. C, Cell invasion was quantified by Matrigel assay following transfection of MDA-231 cells with miR21, anti-miR-21 or appropriate control oligonucleotides. The data are standardized against control, and presented as relative cell invasion numbers. D, Relative miR-21 expression in MDA-435 cells transfected with anti-miR-21 or appropriate control oligonucleotides, determined as in A. E, Relative cell invasion numbers in MDA-435 cells transfected with anti-miR-21 or appropriate control oligonucleotides, as in C. The data are representative of three experiments. *, P < 0.05.

The expression of miR-21 was determined in BCAP-37, MCF-7, MDA-MB-231, and MDA-MB-435 breast cancer cell lines (Fig. 2A). Each breast cancer line expressed elevated levels of miR-21. MDA-MB-231 cells, expressing intermediate levels of miR-21 relative to the other cell lines, were selected to test the impact of modulation of miR-21 expression on invasion using a cell migration assay. Taqman real-time PCR revealed that transfection of miR-21 or anti-miR-21 caused a 2.4-fold increase and 56% decrease of miR-21 expression in MDA-MB-231 cells, respectively, compared to control oligonucleotides (Fig. 2B). While miR-21 overexpression resulted in a 37% increase in cell invasion compared to negative controls (P < 0.05), miR-21 silencing resulted in a 34% decrease in invasive cell number (Fig. 2C; P < 0.05). Similarly, silencing of miR-21 in MDA-MB-435 cells (62% decrease in miR-21 expression, Fig. 2D), which contained the highest baseline miR-21 expression, significantly inhibited cell invasion (48% decrease in invasion, Fig. 2E). Taken together, these data suggest an essential role for miR-21 in tumor cell invasion in vitro.

### TIMP3 protein expression inversely correlates with miR-21 content in breast cancer cell lines

**Figure 3:**
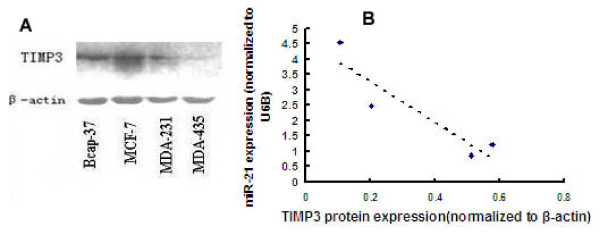
**TIMP3 protein expression correlates with microRNA-21 content in breast cancer cell lines in vitro**. A, Western blot analyses of TIMP3 protein, performed as described in Methods. B, Correlation between miR-21expression and TIMP3 protein levels (Pearson correlation = -0.905; P < 0.05).

As miR-21 regulated TIMP3 expression in glioma and cholangiocarcinoma, we determined baseline TIMP3 protein expression in each of the four breast cancer cell lines relative to miR-21 content (Fig. 3A). In cell lines with high relative miR-21 expression (MDA-MB-435 and MDA-MB-231), a low amount of TIMP3 protein was observed, whereas cell lines with low relative miR-21expression (BCAP-37 and MCF-7) displayed relatively high amounts of TIMP3 protein, resulting in a significant inverse correlation between miR-21 expression and TIMP3 protein content (Fig. 3B; Pearson correlation, r = -0.913, P < 0.05). However, as TIMP3 mRNA expression was very low in each of the four cell lines, a significant correlation between miR-21 and TIMP3 mRNA was not detected (data not shown).

### The TIMP3 3'-UTR is a target for miR-21

**Figure 4:**
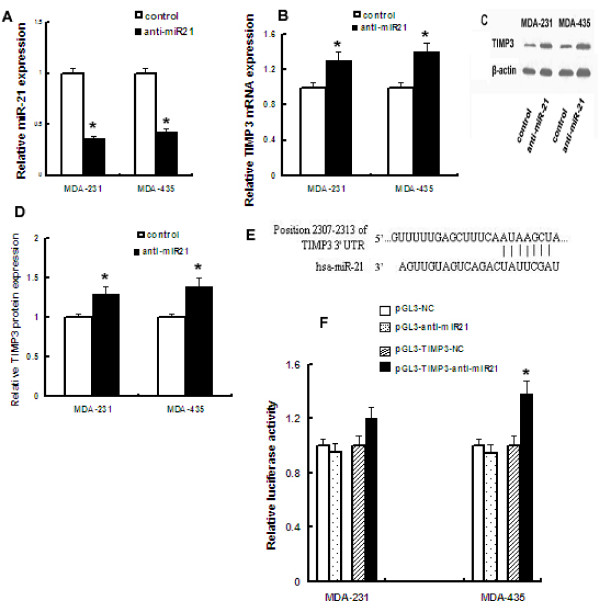
**miR-21 regulates TIMP3 expression at the mRNA and protein level by targeting the 3'untranslated region of TIMP3 mRNA**. A, miR-21 expression was analyzed by TaqMan PCR in MDA-231 and MDA-435 cells following transfection with anti-miR-21 or control oligonucleotides, as in Fig. 2B. B, Relative TIMP3 mRNA expression was analyzed in MDA-231 and MDA-435 cells as described in Methods, following miR-21 silencing as performed in A. C, Western blot analysis of TIMP3 protein expression in MDA-231 and MDA-435 cells following miR-21 silencing as performed in A. D, Quantification of relative TIMP3 protein expression in MDA-231 and MDA-435 cells following miR-21 silencing, as performed in A. E, Generation of cDNA encoding the 3'UTR region of TIMP3 containing a miR-21 binding site. cDNA was subsequently cloned into a Luciferase reporter plasmid. F, Determination of the impact of miR-21 silencing on pGL3-TIMP3 luciferase expression in MDA-231 and MDA-435 cells. Data represents two independent experiments performed in triplicate.*, P < 0.05.

To determine whether suppression of miR-21 impacts TIMP3 transcription, we quantified TIMP3 mRNA in MDA-MB-231 and MDA-MB-435 cells (each expressing high levels of endogenous miR-21) following knockdown of miR-21 expression. Down-regulation of endogenous miR-21 (Fig. 4A) led to a 1.3 and 1.4 fold increase in TIMP3 mRNA in MDA-MB-231 and MDA-MB-435 cells, respectively (Fig. 4B). Similar increases in TIMP3 protein expression following miR-21 knockdown were observed (Fig. 4C, 4D). These data suggest that TIMP3 is regulated by miR-21 in breast cancer cells. In order to determine whether the 3'untranslated region of TIMP3 mRNA is a direct functional target of miR-21, we cloned a 250 bp TIMP3 3'-UTR segment, which includes a potential target site for miR-21 (Fig. 4E), downstream of the pGL3 luciferase reporter gene to generate the pGL3-timp3 vector. This vector was co-transfected into MDA-MB-435 or MDA-MB-231 cell lines together with anti-miR-21 oligonucleotides or miRNA negative control. A renilla luciferase vector (pRL-TK) was used to normalize differences in transfection efficiency. Luciferase activity in MDA-MB-435 cells co-transfected with pGL3-timp3 vector and anti-miR-21 oligonucleotides significantly increased by 38% when compared with negative control (P < 0.05), whereas luciferase activity in MDA-MB-231 cells increased by only 20% (Fig. 4F). These data demonstrate that miR-21 regulates TIMP3 expression at the transcriptional level.

## Discussion

In the present study, we identify increased expression of miR-21 in 78% (25/32) of breast cancer samples analyzed, as compared to patient-matched normal breast epithelium. Further, we identify that the invasive ability of breast cancer cell lines closely correlates with miR-21 expression, as incidence of lymph node metastases increases with miR-21 expression. These data are consistent with reports indicating that miR-21 expression increased with advanced clinical stage and shortened survival of the patients [19], and that miR-21 expression is associated with poor disease-free survival in early stage patients [20].

Greater than 50% of miRNA are located at genomic regions implicated in human cancers, emphasizing the potential importance of miRNA in cancer progression [21]. Specifically, the miR-21 gene is located on chromosome 17q23.2, which is located within the common fragile site FRA17B. This region is frequently found amplified in breast, colon, and lung cancer, consistent with the fact that miR-21 overexpression is widespread in many types of cancer, including the breast [22]. Despite the link of miR-21 to carcinogenesis, little is known regarding the specific mechanism how miR-21 may impact cancer progression.

The correlation of miR-21 expression with tumor metastasis, and supportive evidence that miR-21 regulates cell invasion in vitro, raises the question how miR-21 may impact a cell's metastatic potential. Several factors suggest that miR-21 may be impacting matrix metalloproteinases inhibitors, such as TIMP3, that play a crucial role in cancer invasion and metastasis[23] including recent studies that identified TIMP3 as a functional target of miR-21 in cell invasion and metastasis in glioma and cholangiocarcinoma[15,16]. As TIMP3 expression is down-regulated or lost in several tumor types [24-26], and adenoviral transfer of TIMP3 into HeLa, HT1080 fibrosarcoma, and melanoma cells reduces their invasiveness and stimulates apoptosis[27,28], we tested whether miR-21 may be impacting TIMP3 expression in primary breast cancer specimen as well as four breast cancer-derived cell lines. Our findings report for the first time that microRNA-21 negatively regulates TIMP3 in breast cancer, and suggests that TIMP3 may be negatively regulated by miR-21 at the transcriptional level via binding of the 3'UTR of TIMP3 mRNA. Further, we provide evidence that it is this regulation of TIMP3 expression that impacts cell invasion in vitro. These compelling data support miR-21 regulation of TIMP3 expression as a novel mechanism impacting breast cancer invasion. Our studies suggest that miR-21 regulation of TIMP3 may represent a novel target for therapeutic intervention to prevent breast cancer metastasis, and warrant further investigation.

## Conclusion

Our data identify that miRNA-21 is overexpressed in breast cancer tissues and breast cancer cell lines, promoting breast cancer invasion in multiple cell lines in vitro. Our study suggests that the effect of miRNA-21 expression on cell invasion may be due to the regulation of TIMP3 expression.

## Competing interests

The authors declare that they have no competing interests.

## Authors' contributions

BS and CW carried out oligonucleotide transfection, luciferase report assay; JL, XW and LL contributed to qRT-PCR assay and western blotting analysis; LW, LX and YZ carried out cell culture and migration assay; BS, CW and XS super-vised experimental work and wrote the manuscript. All authors read and approved the final manuscript.
